# Understanding public support for workplace diversity and antidiscrimination policies in Europe

**DOI:** 10.3389/fsoc.2024.1256751

**Published:** 2024-02-23

**Authors:** Lieselotte Blommaert, Marcel Coenders

**Affiliations:** ^1^Department of Sociology, Radboud Social Cultural Research, Radboud University, Nijmegen, Netherlands; ^2^Netherlands Institute for Social Research, The Hague, Netherlands

**Keywords:** workplace diversity policies, antidiscrimination, interventions, public opinion, equity, equal opportunities, inclusion

## Abstract

Societal processes and public opinion can affect whether employers take action and which policy measures they choose to boost diversity, equal opportunities and inclusion, and to reduce discrimination in the workplace. Yet, public opinion regarding workplace diversity initiatives (other than affirmative action) has so far received little scholarly attention, especially in Europe. Consequently, we have very little evidence about how the general public feels about workplace diversity policies – particularly those that are more common or more often discussed in Europe – and about which factors shape public support for these workplace diversity initiatives. Yet, a better understanding of the patterns and antecedents of citizens’ attitudes toward workplace diversity policies is of clear scientific and practical importance. Against this background, this study sheds light on public attitudes toward three different, commonly applied types of workplace diversity policies, and examines which individual-level and – innovatively – national-level conditions shape public support. To do so, we bring together insights from various different and so far largely disconnected strands of research and a range of theoretical perspectives. We use large-scale, representative survey data from two pooled waves of the Eurobarometer, covering 38,009 citizens across 26 European countries. We enrich these data with information on national-level income inequality as well as countries’ labor market and antidiscrimination legislation and policies, obtained from Eurostat and the Migrant Integration Policy Index (MIPEX). Results show significant differences in public support across the three types of policies, with the strongest support for diversity training, followed closely by monitoring recruitment procedures, whereas support for monitoring workforce composition is clearly lower. This is in line with the idea that support tends to be lower for more preferential and prescriptive workplace policies. Furthermore, we find that, in addition to individual-level factors – particularly gender, ethnic minority group membership, personal experiences with discrimination, prejudice, intergroup contact and political orientation – national-level conditions are important antecedents of public support for workplace diversity policies. That is, differences in public attitudes regarding such policies are also shaped by country-level income inequalities, people’s perceptions of how widespread discrimination and unequal opportunities are in society, and national-level laws and policies to fight unequal opportunities.

## 1 Introduction

Over the past years, more and more employers in Europe have adopted policies to boost diversity, equality of opportunities, and inclusion, or to reduce discrimination in the workplace. Such policies can take a variety of forms, including monitoring organizational processes and procedures (to identify and eliminate inequalities, biases or discrimination), formalizing hiring criteria (to minimize the role of biases and discrimination), targeted recruitment (to increase the number of target group members applying for jobs or promotions), training or mentoring programs for target groups (to increase resources and improve opportunities), preferential treatment in selection decisions, voluntary or mandatory training for employees or managers (regarding diversity or avoiding biases), or efforts to increase accountability (e.g., diversity officers or committees) (c.f., Kelly and Dobbin, 1998; Harper and Reskin, 2005; Kalev et al., 2006; Scarborough et al., 2019; Iyer, 2022; Leslie and Flynn, 2022; Bourabain and Verhaeghe, 2023).^1^

Recently, scholars have argued that employers adopt such policies at least partly in response to pressures created by societal changes and movements, such as #MeToo, Black Lives Matter, increased media attention for discriminatory and exclusionary practices, and changing public opinion regarding these issues (e.g., Onyeador et al., 2021; Leslie and Flynn, 2022; Bourabain and Verhaeghe, 2023; Van den Brink and Çelik, 2023). Yet, workplace diversity policies remain controversial. Because not everyone agrees about whether they are needed and suitable, such initiatives – and some types of policies more than others – are regularly subject to debates between advocates and opponents (e.g., Bourabain and Verhaeghe, 2023; Gardberg et al., 2023).

The notion that societal processes and public opinion affect whether employers adopt workplace diversity initiatives is supported by the literatures on policy diffusion (e.g., Maks-Solomon and Drewry, 2021; Gardberg et al., 2023), collective action, solidarity, and allyship (e.g., Saguy et al., 2008; Subasic et al., 2008; Kutlaca et al., 2020; Selvanathan et al., 2020). These literatures stress that social and policy change is more likely to occur when there is broader public support, encouraging those in positions to affect change (in this case employers) to take action. Public resistance and opposition, by contrast, can negatively affect whether employers see workplace diversity policies as necessary and feasible, and hence prevent them from developing or implementing initiatives. Also, we may expect employers to take into account public opinion because their (communication about) diversity policies has been shown to affect their public image among potential applicants and customers (e.g., Jansen et al., 2021; Patel and Feng, 2021; Blommaert and Coenders, 2023).

In this light, it is surprising that public opinion about workplace diversity policies has so far received little scholarly attention, particularly in Europe, where studies on *public* support for such policies are – to our knowledge – so far lacking altogether. That is the case, first of all, because most research on attitudes toward workplace diversity initiatives has until now focused on support among employees and organizational leaders (e.g., Chen and Hooijberg, 2000; Cunningham and Sartore, 2010; Abramovic and Traavik, 2017; Bourabain and Verhaeghe, 2023) or resistance *within organizations* (Thomas, 2008). This is true despite research indicating that, to understand when and why employers adopt diversity policies, we must consider both internal *and* external pressures (e.g., Dobbin et al., 2011; Blommaert and Van den Brink, 2020; Maks-Solomon and Drewry, 2021). Second, whilst public opinion research (in the US) *has* devoted attention to individuals’ attitudes toward affirmative action, “few workplaces use the language of affirmative action to describe their workplace policies [and] the vast majority of them do not fit the prototypical and controversial preferential hiring programs that people may think of when they are asked about affirmative action” (Scarborough et al., 2019, p. 195; see also Bielby, 2000). That is an especially relevant drawback when it comes to understanding public opinion regarding workplace diversity initiatives in the European context, where affirmative action policies are much less common than in the US (Heath et al., 2013; Quillian and Midtbøen, 2021). As a consequence, we know very little about how the general public feels about workplace diversity policies, especially in Europe and when it comes to workplace policies that are more widely used or discussed in the European context.

Against this background, this study sheds light on the extent to which there is public support for workplace diversity policies among European citizens and which determinants play a role in shaping public support. We study support for several types of workplace diversity policies. To explain individual as well as cross-national differences in support, we bring together insights from various different and so far largely disconnected strands of research and a range of theoretical perspectives.

In doing so, we build on and contribute to existing research in several important ways. First, we shed light on public support for workplace diversity policies *in Europe* (using large-scale, representative survey data covering 38,009 citizens across 26 European countries). Existing research on public support for workplace diversity initiatives has (almost) exclusively been conducted in the US. Hence, we so far lack studies focusing on European contexts. This means that it is unclear to what extent outcomes of prior research in this area can be generalized to settings outside the US, including Europe. That problem is amplified by the fact that most research in the US has focused on support for affirmative action policies (c.f., Scarborough et al., 2019). Results pertaining to support for affirmative action cannot simply be translated to the European context because affirmative action initiatives are much less common in Europe (c.f., Heath et al., 2013; Quillian and Midtbøen, 2021) and also because of historical and cultural differences between Europe and the US, for example regarding the role of civil rights movements and implementation of policies in this domain (c.f., Bourabain and Verhaeghe, 2023). To our knowledge, the present study is (among) the first to systematically explore the patterns and determinants of public support for workplace diversity policies in Europe.

Second, we assess whether the factors that have been theorized or found to predict attitudes toward affirmative action and workplace diversity policies in the US are likewise related to support for workplace diversity policies in Europe, but we also build on prior research by examining a broader range of predictors. Studies in this field have traditionally focused on a relatively limited set of potential antecedents of support. Most attention has been paid to factors like demographic group membership (gender and ethnicity/race), and to a lesser extent political ideology, prejudice, personal experience with discrimination, and beliefs about discrimination, inequality or diversity (for literature reviews see: Harper and Reskin, 2005; Harrison et al., 2006; Avery, 2011; Scarborough et al., 2019). In this study, we contribute to the literature by incorporating a wider range of individual-level determinants, including membership of different minority groups, intergroup contact, and sociotropic beliefs about the extent of discrimination and inequality within society (but see, e.g., Scarborough et al., 2019; Iyer, 2022).

Third, very little research has examined the role of contextual factors promoting or decreasing public support for workplace diversity policies. The scarce research that *has* examined the role of contextual factors focused almost exclusively on organizational features (c.f., Harrison et al., 2006; Avery, 2011; Iyer, 2022). Hence, the potential role of (features of) the national context has so far been completely disregarded. This is at least partly due to the fact that existing work in this area has almost exclusively focused on the US and country-comparative research on support for workplace diversity policies is so far lacking. We address this lacuna by theorizing and empirically examining the role of the level of inequality within societies as well as national antidiscrimination and labor market mobility policies and laws. Specifically, we argue that the societal level of inequality is likely to affect the perceived need for workplace diversity policies, while inclusive labor market regulations and policies and anti-discrimination laws and policies affect social norms and raise public awareness of inequality and discrimination (c.f., Müller et al., 2023), which ultimately increases public support for workplace diversity policies.

Finally, we examine public support for three different types of workplace diversity policies, which are commonly applied or discussed: diversity training, monitoring recruitment procedures, and monitoring workforce composition. Diversity or bias trainings are popular among employers, despite evidence showing that they are not (always) particularly effective (e.g., Kalev et al., 2006; Onyeador et al., 2021).^2^ Initiatives to monitor or increase the extent to which recruitment and selection processes offer applicants equal opportunities are often discussed as a promising intervention to limit the effect of interpersonal biases and to boost equality, inclusion and diversity, certainly also in Europe (e.g., Derous and Ryan, 2019; Fibbi et al., 2021; Onyeador et al., 2021). This also applies to monitoring workplace diversity – potentially tied to targets and sanctions when those are not met (e.g., Dobbin et al., 2015; Fibbi et al., 2021). Importantly, studying attitudes toward multiple workplace diversity policies enables us to provide insights in whether and how public attitudes toward these types of policies differ. Research has recently begun to examine differences in attitudes toward various types of policies [see Bourabain and Verhaeghe’s study (2023) on organizational leaders’ attitudes toward policies in higher education and Scarborough and colleagues’ study (2019) on public attitudes toward workplace diversity policies in the US]. Yet, evidence on whether and how levels of public support vary depending on the type of policy is still scarce.

Summarizing, this study aims to answer the following research questions: *(1) to what extent is there public support for or opposition to different types of workplace diversity policies in Europe; (2) which European citizens are more or less likely to support workplace diversity policies and why; (3) how are national-level income inequality and labor market mobility and antidiscrimination laws and policies related to public support for workplace diversity policies?*

## 2 Theoretical background

To explain differences in public support for workplace diversity policies among European citizens, we bring together and build upon a wide range of theoretical perspectives. In this section, we first discuss how public support may vary across different types of workplace diversity policies. Second, we discuss several theoretical perspectives to explain individual differences between citizens in their support for workplace diversity policies. Third, we examine the role of national conditions, namely societal inequality and labor market and anti-discrimination laws and policies.

### 2.1 Differences in public support across different types of workplace diversity policies

Support for workplace diversity policies is expected to vary across different types of initiatives. Although few prior studies have examined differences in support for various workplace diversity policies, the ones that did have indeed found that the level of support varies across different types of policy measures (Scarborough et al., 2019; Bourabain and Verhaeghe, 2023).

One of the most important features of diversity policies – and the one that has received most attention in research on policy support – has to do with “the amount of consideration” that a policy gives to demographic traits (like gender or race/ethnicity) of target groups (Harrison et al., 2006, p. 1014). Policies can be perceived as less fair the more they focus on specific groups based on demographic traits, rather than on merit or qualifications, which are often seen as the factors that should be most relevant for social advancement (Harrison et al., 2006; Scarborough et al., 2019; Bourabain and Verhaeghe, 2023). Public support for policy measures may thus be driven by the perceived fairness of these measures. In line with this argument, Scarborough et al. (2019) found less public support for policy measures where gains for one group may be perceived as coming at the expense of another group, such as targeted recruitment measures. Likewise, Bourabain and Verhaeghe (2023) found more support among European professors for equal opportunity programs in higher education that prioritize merit (while taking group membership into account) compared to preferential programs which target members of marginalized groups. Following Kravitz (1995), Bourabain and Verhaeghe (2023, p. 5) use the labels “non-preferential treatment, preferential treatment and differential or mild preferential treatment programs” to differentiate between workplace policies that vary in this regard. In this approach, preferential policies – unlike non-preferential ones – specifically target members of groups that are underrepresented, disadvantaged, or at risk of discrimination, whereas differential or mild preferential treatment policies only consider group membership when the merit principle is not violated. Drawing on prior theorizing and empirical evidence (Harrison et al., 2006; Scarborough et al., 2019; Bourabain and Verhaeghe, 2023), we expect lower public support for more preferential policy measures, because these can be perceived as more unfair, in the sense that they target specific demographic groups instead of focusing on merit or qualifications.

Another relevant feature of policy measures, which may affect public support, is the degree to which an initiative “forces the hand of, and limits the discretion of” organizational decision makers (Harrison et al., 2006, p. 1014). For instance, some training initiatives may focus merely on raising awareness of bias and inclusion among actors within the organization, whereas other policy measures strongly impact the standard recruitment and hiring processes and thereby limit the agency of decision makers involved in the recruitment and selection process. Harrison et al. (2006) use the term policy ‘prescriptiveness’ to describe this feature of policy measures, whereas others have used ‘policy strength’ (e.g., Slaughter et al., 2002). It is expected that more prescriptive policies generally garner less support and more opposition among the general public. This may be so because such policies can be perceived as violating the merit principle, as Harrison et al. (2006) argue,^3^ or because people generally dislike policies that limit the agency of decision makers to a greater extent, or – in other words – because they dislike policies that are ‘stronger’ or ‘more forceful.’ Such a dislike may result from the fact that more prescriptive policies often require more adaptation of the organizations’ standard practices (they demand a bigger investment), which may lead to resistance, especially in combination with perceptions that changes are being made in order to accommodate the needs of specific (sometimes small) target groups. What may also play a role is that people may perceive more prescriptive policies as reflecting doubts about these decision makers’ morality (e.g., the idea that these policies assume decision makers are racist, sexist, etc.) or their ability (e.g., their ability to make unbiased decisions about job candidates).

Based on the notions that policy support is shaped by the extent to which policy measures are preferential and prescriptive, we expect the level of support to differ across three different workplace policies: (1) *training on diversity* issues for employees and employers; (2) *monitoring recruitment procedures* to ensure that candidates from groups at risk of discrimination have the same opportunities as other candidates with equal skills and qualifications; and (3) *monitoring the composition of the workforce* to evaluate the representation of groups at risk of discrimination. The first type of policy – diversity training – can be considered non-preferential, and the least prescriptive initiative of the three. The second policy – monitoring recruitment procedures – is somewhat more prescriptive. It can be considered to limit the discretion of those who make decisions regarding recruitment and selection at least to a certain extent. Moreover, its stated aim, “to ensure that candidates from groups at risk of discrimination have the same opportunities as other candidates with equal skills and qualifications,” means it specifically targets certain (demographic) groups, but it’s focus on equal skills and qualifications implies that the merit principle is not violated. Hence, we classify this as a differential or mild preferential type of policy. The third and last policy – monitoring workforce composition – can be considered the most prescriptive one out of the three policies. It may be seen as limiting the discretion of those making recruitment and selection decisions to a larger extent. Given that there is no mention of equal skills and qualifications here, we may even classify this as a preferential policy. Also, it refers to the (under)representation of specific (demographic) groups in the workforce and could perhaps be perceived as moving in the direction of – or foreshadowing – quota policies, which constitute a stronger form of preferential policy. As such, we expect less support among the general public for monitoring workforce composition, compared to monitoring recruitment procedures and diversity training, and less support for monitoring recruitment procedures than for diversity training (*hypothesis 1*).

### 2.2 Differences between European citizens in public support for workplace diversity policies

In this section, we bring together several theoretical perspectives to explain public support for workplace diversity policies. We build on previous research examining public support for affirmative action policies or governmental minority targeted action in general (e.g., Sniderman and Piazza, 1993; Kravitz, 1995; Harrison et al., 2006), support among employees or the general public for diversity in organizations (e.g., Avery, 2011; Scarborough et al., 2019), as well as related research on outgroup prejudice and intergroup relations (e.g., Bobo, 1998; Stephan et al., 2002). By combining various theoretical perspectives from different fields, we build an elaborate theoretical framework to understand public support for workplace diversity policies.

We distinguish three types of reasons to support workplace diversity policies. First, public support or opposition may be driven by interest (Sears and Funk, 1991; Harrison et al., 2006; Avery, 2011; Scarborough et al., 2019; Bourabain and Verhaeghe, 2023). Citizens assess the potential beneficiaries of organizational policies, and take into account the expected personal gains or losses. Second, citizens may support or oppose such policies because they have certain ideological beliefs, values, and attitudes toward or experiences with beneficiary target groups, such as political-ideological stances on equality and intergroup attitudes (Harrison et al., 2006; Avery, 2011; Bourabain and Verhaeghe, 2023). A related theoretical approach focuses on sociotropic considerations, i.e., perceptions of the treatment and opportunities of different groups within society. According to this third approach, public support or opposition for diversity policies may not only stem from tangible self-interest or individual ideological or attitudinal dispositions, but can also be influenced by citizens’ assessment of the societal obstacles and opportunities for certain groups within society, which inform the perceived need for (stronger) diversity policies.

According to self-interest theory (Sears and Funk, 1991), citizens support policies when it is (perceived to be) in their own best interest to do so. In line with rational choice theory, citizens are considered here as rational actors (Monroe, 1991) that assess the potential personal benefits or losses of the policy initiative. Although self-interest is often considered an important mechanism in explaining policy support, the empirical evidence is not always that strong, as was already shown by classic studies such as the one by Sears et al. (1980). Chong et al. (2001) argued that self-interest matters more when people actually have a stake in a policy and can recognize this as such.

One of the indicators for such a stake in diversity policies is minority group membership. Minority or underrepresented groups are expected to be more in favor of policies that foster diversity, inclusion and equal opportunities, and counter discrimination. Some evidence from the US also showed that people – regardless of their racial in-group – tend to associate the concept of diversity more with (racial) minorities than with Whites (Unzueta and Binning, 2010). In line with these notions, research in the US indeed showed that support for affirmative action policies is much higher among Blacks than Whites (e.g., Schuman et al., 1997). Likewise, Scarborough et al. (2019) found that the level of support for workplace policies targeted at racial minorities was highest among Blacks, followed by Latinos, with Whites showing the lowest support. Similarly, this study showed that support for workplace policies aimed at supporting women was higher among women than men. In the present study, we take into account the role of gender as well as self-identification as a member of a (range of different) minority groups. Hence, our first hypothesis based on the logic of self-interest reads: public support for workplace diversity policies is stronger among (h2a) women and (h2b) those who consider themselves a member of a minority group (*hypothesis 2*).

Based on the self-interest logic one would also expect that those who have experienced being discriminated against based on their group membership, are more in favor of workplace diversity policies that foster diversity and remedy discriminatory practices (Kravitz et al., 2006). Hence, we expect that public support for diversity policies is stronger among those who have personally experienced discrimination (*hypothesis 3*). As there are large differences in (actual) hiring discrimination between ethnic majority and minority groups (e.g., Koopmans et al., 2019; Lancee, 2021; Lippens et al., 2023), personal experiences with discrimination are likely to partly explain why ethnic minority groups would be more in favor of diversity policies.

Whereas the former account focuses on interests and the expected personal gains or losses of diversity policies, an alternative theoretical approach focuses on ideological beliefs, values, and attitudes toward or experiences with policies’ target groups as drivers for public support for diversity policies.^4^ In this context, outgroup prejudice is one factor that is considered important for explaining public support for such policies (c.f., Schuman et al., 1997). Specifically, opposition toward these policies is thought to stem from prejudices toward the perceived beneficiary target group. According to outgroup threat theories (for a meta-analysis see Riek et al., 2006), such prejudices are driven by a perceived conflict of group interest: the more people perceive that their group position is threatened by an outgroup, the higher their level of prejudice (e.g., Bobo, 1998; Scheepers et al., 2002). Perceived outgroup threat and prejudice can therefore increase opposition to workplace policies that improve workplace and career opportunities for the outgroup (e.g., Konrad and Hartmann, 2001). In line with these notions, research in the US found clear effects of different types of outgroup threat on opposition toward workplace diversity or affirmative action policies (e.g., Renfro et al., 2006). Based on this, we expect that public support for workplace diversity policies is higher among individuals who are less prejudiced (*hypothesis 4*).

Outgroup contact has been shown to reduce prejudice (see for a review: Pettigrew and Tropp, 2011). According to contact theory (Allport, 1954; Brown and Hewstone, 2005) contact with outgroup members reduces outgroup anxiety and increases empathy. Typically, these effects generalize beyond the immediate contact situation to other situations and even other outgroups (Pettigrew and Tropp, 2011). We argue that, through increased empathy and perspective taking, outgroup contact can not only reduce prejudice, but may also directly increase support for policies that boost equal opportunities and treatment of outgroups. Indeed, Tropp and Barlow (2018) argue that intergroup contact (between racial groups) is one of the most promising pathways to make advantaged groups acknowledge and care about inequality. Hence, we expect that public support for workplace diversity policies is higher among those who have more minority friends and acquaintances (*hypothesis 5*).

Public support for diversity policies may also stem from political-ideological dispositions, like views about the desired and legitimate degree of societal inequality, anti-egalitarianism and individual differences in social dominance orientation (Pratto et al., 1994). Left-wing oriented persons tend to prefer less inequality (Alesina and Giuliano, 2009) and have therefore been theorized and found to be more supportive of diversity policies that foster equal opportunities and inclusion (e.g., Sniderman and Carmines, 1997; Scarborough et al., 2019). Hence, we expect that public support for diversity policies is higher among those with a more left-wing political orientation (*hypothesis 6*). We note that political orientation is not only predictive of public opinion on socio-economic issues, but is also relevant for socio-cultural issues, as political orientation is interrelated with cultural conservatism, group identity and prejudice. Hence, the theoretical distinction between prejudice and political orientation as two distinct determinants of public support for diversity policies may be conflated. Nevertheless, in debates on affirmative action and inclusive policies, the role of political-ideological factors (such as political orientation) is often considered separately from the role of prejudice. That is, some argue that public opposition does not stem from prejudice or threat from minorities, but rather from ‘race-neutral,’ ideological views regarding economic egalitarianism and meritocratic ideals (Sniderman and Piazza, 1993). However, seemingly race-neutral arguments can also be used as justifications for preservation of ingroup dominance, and outgroup prejudice or threat. For example, O’Brien et al. (2010) found that White Americans more strongly endorse the race-neutral objection that affirmative action harms its intended beneficiaries (by undermining their self-esteem) if they believe that affirmative action harms the interests of Whites.

Next, we take into account the role of beliefs regarding societal-level inequality and discrimination. Awareness of these societal problems can serve as justifications for social and policy change (e.g., Kluegel and Smith, 1986; Scarborough et al., 2019; Settele, 2022). There are marked differences between individuals in their assessment of the opportunity structure within society [see for instance Müller et al. (2023) on beliefs about the prevalence of discrimination in Europe]. In contrast to the aforementioned self-interest or group-interest motivations, such beliefs are more related to sociotropic motivations, emphasizing ‘what is good for the society?’ instead of ‘what is good for me?’ (Lockerbie, 2006). The more citizens are convinced that inequality and discrimination exist, the more they may regard diversity policies as a justified means to remedy these obstacles for the inclusion of minority groups. Although research on such matters is still scarce, some recent studies provided empirical evidence supporting this logic. Haaland and Roth (2023) found that citizens’ beliefs about the extent of racial discrimination in the US were strongly related to donations to a pro-black civil rights organization and support for pro-black policies. Uluğ and Tropp (2021) found that witnessing incidents of racial discrimination increased engagement for collective action for racial justice, through enhanced awareness of racial privilege. Mijs et al. (2023) found that in the Netherlands (not the US) there was a marginally significant effect of providing evidence of ethnic and racial inequalities on participants’ belief that government bears responsibility for fighting discrimination. Based on this, we expect that public support is positively related to the degree to which citizens believe that (h7a) discrimination of minority groups is widespread and (h7b) there are unequal opportunities in hiring in their country (*hypothesis 7*).

### 2.3 The role of the national context

Previous research on support for diversity policies has neglected the potential role of the societal context. We address this lacuna by examining two relevant features of the national context. First, in line with the aforementioned reasoning, we examine the role of the actual degree of inequality within society. In societies with more unequal opportunity structures, citizens may perceive a stronger need for policy initiatives that aim to foster equal opportunities and inclusion, and counter discrimination (Kluegel and Smith, 1986; Scarborough et al., 2019; Settele, 2022; Haaland and Roth, 2023; Mijs et al., 2023). Indeed, the OECD (2021) found that actual income inequality in a region was correlated with stronger preferences for redistribution among the general public, and furthermore, that changes in national inequality correspond with changes in public demand for redistribution. Hence, we hypothesize that the higher the income inequality within a nation is, the more the public will support workplace diversity initiatives (*hypothesis 8*).

Second, we take into account the role of national-level legislation and policies. National laws and policies and public opinion may be linked in various complex and dynamic ways (Bilgili et al., 2015; Callens and Meuleman, 2017). Different theoretical perspectives point to opposing causal directions, and in reality the link is likely reciprocal. According to policy responsiveness theory, policymakers respond to public preferences (e.g., Brooks and Manza, 2006). In contrast, according to policy feedback theory, policies reshape the political environment and the broader societal context and influence public attitudes (Mettler and Soss, 2004). Drawing on the latter perspective, the present study derives predictions from theoretical approaches that focus on political and public agenda setting mechanisms, shaping group membership, identities and interests, and framing problem definitions and rationales (see, e.g., Mettler and Soss, 2004; Larsen, 2019). Such theoretical approaches argue that policies can influence public “beliefs about what is possible, desirable, and normal” (Soss and Schram, 2007, p. 213). Furthermore, to derive expectations on the link between national-level policies and public support for workplace diversity policies, we focus on two important legislation and policy domains. First, we examine the role of national-level anti-discrimination legislation and policies. Stronger anti-discrimination laws and policies likely raise public awareness of the problem of discrimination and enforce social norms. In line with this idea, Ziller (2014) found that in countries with stronger anti-discrimination laws and policies, citizens are more likely to be aware of discrimination as a societal problem and tend to have greater knowledge about their rights related to equal treatment and discrimination. Second, we take into account national labor market policies. We expect that more inclusive national policies that foster equal opportunities for labor market participation create more public awareness of the need to improve inclusion, diversity and equal opportunities at the workplace. Therefore, our final hypothesis is: the more inclusive the labor market policies (h9a) and the more comprehensive anti-discrimination laws and policies (h9b) are, the higher the public support for workplace diversity policies (*hypothesis 9*). Table 1 summarizes our hypotheses.

**Table 1:** Summary of hypotheses.

H#	Independent variables	Expected relationship to support
*Policy type*		
H1	Monitoring recruitment procedures (versus diversity training) Monitoring workforce composition (versus diversity training)	− −−
*Individual-level factors*		
H2	Women (versus men) Minority group members (versus majority)	+ +
H3	Personal experience with discrimination (versus none)	+
H4	Outgroup prejudice	−
H5	Minority friends or acquaintances	+
H6	Right-wing political orientation	−
H7	Belief that Discrimination is widespread in country There are unequal opportunities in hiring in country	+ +
*Country-level factors*		
H8	Country-level income inequality	+
H9	Country-level laws and policies for An inclusive labor market Antidiscrimination	+ +

## 3 Data and measurement

### 3.1 Data

To test our hypotheses, we use survey data from two (pooled) waves of the Eurobarometer (EB), enriched with information on national income inequality and national antidiscrimination and labor market mobility policies and laws. The EB survey is, to our knowledge, the only cross-national survey that measures the general public’s attitudes toward workplace policies that employers can implement to foster diversity and equal opportunities and to prevent discrimination. The sample design applied in all European Union member states is a multi-stage, random sample of the resident population aged 15 years and over. Interviews were conducted face-to-face in respondents’ home (European Commission, 2012, 2015).

Support for workplace diversity policies was measured in the 2012 and 2015 rounds of the special EB-surveys ‘Discrimination in the European Union’ [EB 77.4 (European Commission and European Parliament, 2015) and EB 83.4 (European Commission, 2018)]. We therefore use pooled data from these two rounds, which cover 29 and 30 countries, respectively. Great Britain and Northern Ireland were surveyed separately in these rounds, but we combined them to be able to enrich them with contextual data. We excluded Croatia because it was not covered in the 2012 EB round. Furthermore, we excluded Malta because no comparable information on national income inequality was available. This led to a dataset with information on 53,337 respondents in 26 European countries, each covered in both years. These countries are: Austria, Belgium, Bulgaria, Cyprus, Czech Republic, Denmark, Estonia, Spain, Finland, France, Germany, Greece, Hungary, Ireland, Italy, Lithuania, Latvia, Luxembourg, the Netherlands, Poland, Portugal, Romania, Sweden, Slovenia, Slovakia, and United Kingdom.

For our analyses, we included those respondents with valid answers to the items used to measure our three dependent variables (*n* = 45,094). Respondents with missing values on any of the independent variables in our analyses were listwise deleted, unless stated otherwise below. This resulted in an analytical sample of 38,009 respondents in 26 countries.

### 3.2 Measurement

#### 3.2.1 Dependent variables

Our dependent variables – gauging support for different workplace policies to promote diversity and equity and combat discrimination^5^ – were measured using three questions asking “To what extent do you support or oppose each of the following measures in the workplace to foster diversity?: (1) Training on diversity issues for employees and employers; (2) Monitoring the composition of the workforce to evaluate the representation of groups at risk of discrimination; (3) Monitoring recruitment procedures to ensure that candidates from groups at risk of discrimination have the same opportunities as other candidates with equal skills and qualifications.” Respondents could answer “totally oppose” (0), “somewhat oppose” (1), “somewhat support” (2), or “totally support” (3). Hence, higher scores indicate more support. We treat these variables as linear.

#### 3.2.2 Individual-level independent variables

We include several individual-level independent variables. To assess the role of self-interest, we take into account *gender* (0 = male, 1 = female), and 5 dichotomous variables indicating whether respondents consider themselves to be part of one or more minority group(s), or not. The latter was measured by asking respondents: “Where you live, do you consider yourself to be part of any of the following?: (1) an *ethnic minority*, (2) a *religious minority*, (3) a *sexual minority* (like being gay, lesbian, bisexual, transgender or transsexual), (4) a *minority in terms of disability*, (5) any *other minority* group.” Another variable that we included to assess the role of self-interest measures whether someone has *personally experienced discrimination* or not. Respondents were asked whether, in the 12 months preceding data collection, they had personally felt discriminated against or harassed on the following grounds: (1) ethnic origin, (2) gender, (3) sexual orientation (being gay, lesbian or bisexual), (4) being over 55 years old, (5) being under 30 years old, (6) religion or beliefs, (7) disability, (8) gender identity (being transgender or transsexual), and (9) for another reason. We constructed a dichotomous variable indicating whether a respondent personally felt discriminated based on at least one of these different grounds (1) or had not felt discriminated (0).^6^

To measure *intergroup contact*, respondents were asked whether they had friends or acquaintances (answer categories ‘yes’ or ‘no’) who are: (1) of a different ethnic origin than the respondent, (2) Roma, (3) gay, lesbian or bisexual, (4) disabled, (5) of a different religion or who have different beliefs than the respondent, and (6) transgender or transsexual. We added up respondents’ scores on these questions, constructing an indicator of the extent to which respondents have intergroup contact (0–6), with higher scores expressing that one has friends or acquaintances belonging to a larger number of different outgroups. To assess the role of *outgroup prejudice*, we calculated respondents’ average score across 8 items asking them: “Using a scale from 1 to 10, please tell me how you would feel about having a person from each of the following groups in the highest elected political position in [country]: (1) a woman, (2) a gay, lesbian or bisexual person, (3) a person from a different ethnic origin than the majority of the population, (4) a person under 30 years old, (5) a person from a different religion than the majority of the population, (6) a person with a disability, (7) a person over 75 years old, (8) a transgender or transsexual person.” Answer categories ranged from (1) ‘Not at all comfortable’ to (10) ‘Totally comfortable,’ but were recoded such that higher scores express higher levels of prejudice. To include *political left–right self-placement*, we used the item “In political matters people talk of ‘the left’ and ‘the right.’ How would you place your views on this scale?” (1 = most left; 10 = most right). Relatively many respondents (*n* = 8,267) did not provide valid answers to this question. Rather than excluding these and loosing this relatively large (and possibly selective) group of respondents, we assigned them the average score on this item in the respective country-year combination and included a dichotomous variable indicating whether respondents provided valid answers to this item.

Next, two variables refer to citizens’ beliefs about the prevalence of discrimination and unequal hiring opportunities in their country. To measure *beliefs about the prevalence of discrimination in their country*, we calculated the average score on 8 items asking respondents: “For each of the following types of discrimination, could you please tell me whether, in your opinion, it is very widespread, fairly widespread, fairly rare or very rare in [country]?: discrimination based on (1) ethnic origin, (2) sexual orientation (being gay, lesbian or bisexual), (3) being over 55 years old, (4) being under 30 years old, (5) religion or beliefs, (6) disability, (7) gender identity (being transgender or transsexual), and (8) gender.” In addition to the answer categories mentioned in the question, the interviewer could write down “non-existent” if respondents gave this answer spontaneously. As few people did so, and the tendency to give such answers spontaneously may be influenced by cultural, personal, and interviewer factors, we combined this category with “very rare.” Scores therefore range from (0) ‘very rare or non-existent’ to (3) ‘very widespread.’ We operationalized respondents’ *beliefs about unequal opportunities in hiring* in their country using 14 items asking: “In [country] when a company wants to hire someone and has the choice between two candidates with equal skills and qualifications, which of the following criteria may, in your opinion, put one candidate at a disadvantage?: a candidate’s “name,” “address,” “way of speaking, his or her accent,” “skin color or ethnic origin,” “gender (male or female),” “gender identity (being transgender or transsexual),” “sexual orientation (being gay, lesbian or bisexual),” “age, if he or she is over 55 years old,” “age, if he or she is under 30 years old,” “a disability,” “the expression of a religious belief (e.g., wearing a visible religious symbol),” “look (manner of dress or presentation),” “physical appearance (size, weight, face, etc.),” and “other.” We constructed a variable indicating the extent to which respondents believe unequal opportunities in hiring (based on different grounds) exist by counting the number of criteria that put a candidate at a disadvantage according to the respondent. We capped the maximum score at 10 to avoid that the relatively few respondents with higher scores than that had a disproportionate impact on our estimates.

#### 3.2.3 Macro-level independent variables

At the macro-level, we included a measure of *income inequality* in the respective countries (Gini index), for which we obtained information from Eurostat (2021). The index can – theoretically – range from 0% (perfect equality) to 100% (maximum inequality). Across the 26 countries in our analyses, scores range from 23.8 (in Slovenia) to 35.6 (in Estonia). Furthermore, to measure national regulations and policies aimed at facilitating labor market equality and combatting discrimination, we used figures from the Migrant Integration Policy Index (MIPEX) project. MIPEX offers a rich, comprehensive and multi-dimensional assessment of laws and policies to increase equality and opportunities for migrants to participate in society.^7^ We include indicators of *antidiscrimination policies and laws* and *labor market mobility policies and laws.* Country-year specific scores come from two policy domain-specific sub-indices of the MIPEX, using the MIPEX 2015 database (Huddleston et al., 2015).^8^ These scores are based on country-expert ratings of national antidiscrimination and labor market mobility laws and policies. They are available as a yearly time series. Compared to other integration policy indices, MIPEX has the widest coverage in terms of countries and regions, but also policy areas and indicators, as well as the most robust data collection method, using objective policy categorizations by national experts. Moreover, MIPEX is the most widely and intensively used index in quantitative research (c.f., Bilgili et al., 2015). The indices (theoretically) range from 0 to 100, with higher values indicating more comprehensive laws and policies to facilitate participation and inclusion of migrants. Across the 26 countries in our analyses, there are substantial variations in terms of both antidiscrimination policies (scores range from 32 in Estonia to 89 in Bulgaria) and labor market mobility policies (scores range from 21 in Slovakia to 98 in Sweden). All macro-level independent variables were matched with a one year lag prior to the EB survey data,^9^ to minimize concerns about causality as best we could with the available (cross-sectional) data.

#### 3.2.4 Control variables

At the individual level, we first of all controlled for respondents’ *educational level*, measured as the age at which they completed their education. For those who were still studying, we used their age at the time of data collection as a proxy. To prevent outliers, values for respondents who were younger than 14 when they completed their education were set to the cut-off value of 13 and the maximum age for finishing education was capped at 26. To enhance interpretability, the scores were subtracted by 13 in order to create a variable with values ranging from 0 to 13. Moreover, we controlled for respondents’ *social position*, based on a question about respondents’ current occupation or activities. We combined the 18 categories distinguished in the EB into six categories. Firstly, we distinguished between those with a paid job, those who are unemployed, and those who are not active in the labor market (including students, retired people, those who are unable to work, and those who are active within their own housework and childcare). Secondly, for those with a paid job, we distinguished between manual workers, routine non-manual workers, self-employed and small employers, and those working in the service class. We also controlled for *age* (we subtracted age in years with 15 in order to create a variable with a minimum value of 0) and the *urbanization* level of respondents’ place of residence (rural area or village, small or middle sized town, large town). Lastly, as prosocial behavior may also be related to religious beliefs and communities (Graham and Haidt, 2010), we controlled for *religious denomination* (“non-believer/atheist/agnost,” “catholic,” “other Christian,” “other religion”).

At the macro level, we controlled for the GDP and the share of the population that was foreign-born in the respective country-years. Information on *GDP per capita* (PPP) was obtained from the World Bank (2021) and was divided by 1,000 for the analyses. Information on the foreign-born population was obtained from Eurostat (2023) and measured as the percentage of residents who are born abroad. Like the other macro-level variables, these two control variables were included with a one year time lag prior to the year of survey data collection. Descriptive statistics for all variables in our analyses are displayed in Table 2. Correlations between the macro-level variables are presented in Table 3.

**Table 2:** Descriptive statistics independent variables.

	Mean/proportion	Std. dev.	Min	Max	
*Individual-level independent variables*					
Gender (Female)	0.545	0.498	0	1	
Ethnic minority	0.042	0.200	0	1	
Religious minority	0.041	0.197	0	1	
Sexual minority	0.012	0.109	0	1	
Disability minority	0.026	0.158	0	1	
Other minority	0.020	0.141	0	1	
Personally experienced discrimination	0.179	0.383	0	1	
Outgroup prejudice	4.374	2.176	1	10	
Intergroup contact	2.610	1.598	0	6	
Political left–right placement	5.227	2.076	1	10	
*National-level conditions*					
Antidiscrimination laws & policies	58.715	18.868	21	98	
labor market integration laws & policies	64.802	16.106	32	89	
Income inequality (GINI, lagged)	29.998	3.622	23.8	35.6	
Belief prevalence discrimination	2.234	0.708	0	4	
Belief unequal opportunities hiring	4.223	2.753	0	10	
*Individual-level control variables*					
Age	48.585	17.822	15	95	
Education	19.117	3.637	13	26	
Occupational class					
Unemployed	0.081	0.273	0	1	
Nonactive	0.409	0.492	0	1	
Manual worker	0.121	0.326	0	1	
Routine non-manual worker	0.205	0.404	0	1	
Self-employed and small employers	0.039	0.194	0	1	
Service class	0.146	0.353	0	1	
Religious denomination					
Non-believer	0.219	0.413	0	1	
Catholic	0.410	0.492	0	1	
Other Christian	0.341	0.474	0	1	
Other denomination	0.030	0.172	0	1	
Urbanization	0.039	0.194	0	1	
Rural	0.314	0.464	0	1	
Small or middle sized town	0.407	0.491	0	1	
Large town	0.279	0.449	0	1	
*National-level control variables*					
GDP *per capita* (lagged)	39.843	14.522	18.023	108.761	
Foreign-born population in % (lagged)	10.524	6.432	0.906	43.270	

Sources data: EB 77.4 & 83.4 (individual-level), MIPEX (national-level policies), Eurostat (GINI, foreign-born population), Worldbank (GDP); n-individuals: 38,009, n-country-years: 52.

**Table 3:** Correlations between macro-level variables.

	(1)	(2)	(3)	(4)	(5)
(1) Income inequality	1				
(2) Labor market policies	−0.014	1			
(3) Antidiscrimination policies	−0.177	0.188	1		
(4) GDP *per capita*	−0.398^*^	0.169	−0.085	1	
(5) Foreign-born population	−0.076	0.024	−0.304^*^	0.778^*^	1

*p < 0.05. Sources data: MIPEX (national-level policies), Eurostat (GINI, foreign-born population), World Bank (GDP); n-country-years: 52.

## 4 Analyses and results

### 4.1 Support for different types of workplace diversity policies

Table 4 shows the mean scores for public support for the three different workplace diversity policies. The mean scores are all somewhat above 2, which falls between the categories “somewhat support” (2) and “totally support” (3), indicating that the general public is rather supportive of these policies. However, there are clear differences in support across the three types of policies: support is highest for diversity training (2.243), followed closely by monitoring recruitment procedures (2.230). Support for monitoring workforce composition is clearly lower (2.047) than for both other policy types. Results from mean-comparison t-tests confirm that all these differences are statistically significant (*p* < 0.05), which provides support for *hypothesis 1*.

**Table 4:** Mean scores public support for three types of workplace policies.

	Mean	Std. dev.	Min	Max	
Support for diversity training	2.243	0.777	0	3	
Support monitoring recruitment procedures	2.230^a^	0.809	0	3	
Support monitoring workforce composition	2.047^ab^	0.855	0	3	

Significance: ^a^ differs significantly from mean support for diversity training, ^b^ differs significantly from mean support for monitoring recruitment procedures. Sources data: EB 77.4 & 83.4; n-individuals: 38,009, n-country-years: 52.

### 4.2 Multilevel analyses

To empirically test our other hypotheses, we estimated linear mixed-effects multilevel models.^10^ This enables us to account for the hierarchical nature of our data – with individual respondents (level 1, *n* = 38,009) nested in country-year combinations (level 2, *n* = 52) – and to accurately estimate relationships between individual-level and macro-level variables.

Analyses regarding our three dependent variables – support for diversity training, monitoring recruitment procedures, and monitoring workforce composition – are presented in Tables 5–7, respectively.

**Table 5:** Support for diversity training (model 1 to 3).

	Model 1		Model 2		Model 3	
	b	se	b	se	b	se
*Individual-level variables*						
Gender (Male = ref)	0.105^***^	(0.008)	0.103^***^	(0.008)	0.085^***^	(0.008)
Ethnic minority	0.052^**^	(0.020)	0.043^*^	(0.020)	0.034	(0.020)
Religious minority	−0.024	(0.020)	−0.031	(0.020)	−0.023	(0.020)
Sexual minority	−0.008	(0.036)	−0.018	(0.036)	−0.047	(0.035)
Disability minority	0.038	(0.025)	0.025	(0.025)	0.021	(0.024)
Other minority	0.027	(0.027)	0.016	(0.027)	0.008	(0.027)
Experienced discrimination			0.047^***^	(0.010)	0.044^***^	(0.010)
Outgroup prejudice					−0.048^***^	(0.002)
Intergroup contact					0.030^***^	(0.003)
Left–right placement					−0.019^***^	(0.002)
Missing left–right placement					0.006	(0.011)
*Individual-level control variables*						
Age	−0.001^***^	(0.000)	−0.001^***^	(0.000)	−0.001^*^	(0.000)
Education	0.007^***^	(0.001)	0.007^***^	(0.001)	0.003^**^	(0.001)
Social position (service class = ref)						
Unemployed	0.028	(0.018)	0.022	(0.018)	0.037^*^	(0.017)
Non-Active	0.035^**^	(0.013)	0.033^**^	(0.013)	0.053^***^	(0.013)
Manual worker	−0.014	(0.016)	−0.015	(0.016)	0.006	(0.016)
Self-employed & small employers	−0.029	(0.023)	−0.030	(0.023)	−0.001	(0.022)
Routine non-manual workers	−0.010	(0.014)	−0.010	(0.014)	0.003	(0.013)
Denomination (Non-believer = ref)						
Catholic	−0.006	(0.012)	−0.005	(0.012)	0.026^*^	(0.012)
Other Christian	0.031^*^	(0.013)	0.031^*^	(0.013)	0.062^***^	(0.013)
Other denomination	0.073^**^	(0.024)	0.070^**^	(0.024)	0.078^**^	(0.024)
Urbanization (Rural = ref)						
Small or middle sized town	−0.008	(0.009)	−0.010	(0.009)	−0.013	(0.009)
Large town	−0.012	(0.010)	−0.014	(0.010)	−0.025^*^	(0.010)
Constant	2.174^***^	(0.034)	2.168^***^	(0.034)	2.372^***^	(0.035)
sd(CountryYear)	0.040^***^	(0.008)	0.040^***^	(0.008)	0.032^***^	(0.006)
sd(Residual)	0.557^***^	(0.004)	0.557^***^	(0.004)	0.543^***^	(0.004)
Observations	38,009		38,009		38,009	
*Notes*: *p < 0.05, **p < 0.01, ***p < 0.001 (two-tailed). Sources data: EB 77.4 & 83.4 (individual-level), MIPEX (national-level policies), Eurostat (GINI and percentage foreign born), World Bank (GDP); n-individuals: 38,009, n-country-years: 52. All macro variables are lagged (one year) and centered on the grand mean.						

Table 5 (Continued). Support for diversity training (model 4 to 5).				
	Model 4		Model 5	
	b	se	b	se
*Individual-level variables*				
Gender (Male = ref)	0.086^***^	(0.008)	0.071^***^	(0.008)
Ethnic minority	0.034	(0.020)	0.044^*^	(0.019)
Religious minority	−0.023	(0.020)	−0.027	(0.020)
Sexual minority	−0.048	(0.035)	−0.059	(0.035)
Disability minority	0.021	(0.024)	0.010	(0.024)
Other minority	0.009	(0.027)	0.001	(0.027)
Experienced discrimination	0.044^***^	(0.010)	0.010	(0.010)
Outgroup prejudice	−0.048^***^	(0.002)	−0.051^***^	(0.002)
Intergroup contact	0.030^***^	(0.003)	0.024^***^	(0.003)
Left–right placement	−0.019^***^	(0.002)	−0.016^***^	(0.002)
Missing left–right placement	0.005	(0.011)	0.011	(0.011)
*National-level conditions*				
Income inequality (GINI)	0.026^***^	(0.006)	0.029^***^	(0.006)
Antidiscrimination policies	0.003^*^	(0.001)	0.002	(0.001)
Labor market integration policies	0.003^*^	(0.001)	0.002	(0.001)
Belief prevalence discrimination			0.118^***^	(0.006)
Belief unequal opportunities hiring			0.015^***^	(0.002)
*Individual-level control variables*				
Age	−0.001^*^	(0.000)	−0.000	(0.000)
Education	0.003^**^	(0.001)	0.003^*^	(0.001)
Social position (service class = ref)				
Unemployed	0.037^*^	(0.017)	0.036^*^	(0.017)
Non-Active	0.054^***^	(0.013)	0.055^***^	(0.013)
Manual worker	0.007	(0.016)	0.012	(0.016)
Self-employed & small employers	−0.001	(0.022)	0.004	(0.022)
Routine non-manual workers	0.003	(0.013)	0.006	(0.013)
Denomination (Non-believer = ref)				
Catholic	0.025^*^	(0.012)	0.035^**^	(0.012)
Other Christian	0.062^***^	(0.013)	0.071^***^	(0.012)
Other denomination	0.077^**^	(0.024)	0.089^***^	(0.024)
Urbanization (Rural = ref)				
Small or middle sized town	−0.013	(0.009)	−0.014	(0.009)
Large town	−0.025^*^	(0.010)	−0.031^**^	(0.010)
*National-level control variables*				
GDP *per capita*	−0.002	(0.002)	−0.002	(0.002)
Foreign-born population (%)	0.011^*^	(0.004)	0.009^*^	(0.004)
Constant	2.367^***^	(0.031)	2.049^***^	(0.034)
sd(CountryYear)	0.016^***^	(0.003)	0.017^***^	(0.003)
sd(Residual)	0.543^***^	(0.004)	0.534^***^	(0.004)
Observations	38,009		38,009	

*Notes*: ^*^p < 0.05, ^**^p < 0.01, ^***^p < 0.001 (two-tailed). Sources data: EB 77.4 & 83.4 (individual-level), MIPEX (national-level policies), Eurostat (GINI and percentage foreign born), World Bank (GDP); n-individuals: 38,009, n-country-years: 52. All macro variables are lagged (one year) and centered on the grand mean.

**Table 6:** Support for monitoring recruitment procedures (model 1 to 3).

	Model 1		Model 2		Model 3	
	b	se	b	se	b	se
Gender (Male = ref)	0.118^***^	(0.008)	0.116^***^	(0.008)	0.098^***^	(0.008)
Ethnic minority	0.049^*^	(0.021)	0.039	(0.021)	0.030	(0.020)
Religious minority	−0.019	(0.021)	−0.027	(0.021)	−0.019	(0.021)
Sexual minority	−0.012	(0.037)	−0.023	(0.037)	−0.053	(0.037)
Disability minority	0.047	(0.026)	0.033	(0.026)	0.029	(0.025)
Other minority	−0.031	(0.028)	−0.043	(0.029)	−0.050	(0.028)
Experienced discrimination			0.052^***^	(0.011)	0.050^***^	(0.011)
Outgroup prejudice					−0.050^***^	(0.002)
Intergroup contact					0.028^***^	(0.003)
Left–right placement					−0.022^***^	(0.002)
Missing left–right placement					0.006	(0.011)
*Individual-level control variables*						
Age	−0.003^***^	(0.000)	−0.003^***^	(0.000)	−0.002^***^	(0.000)
Education	0.006^***^	(0.001)	0.006^***^	(0.001)	0.002	(0.001)
Social position (service class = ref)						
Unemployed	0.064^***^	(0.018)	0.057^**^	(0.018)	0.072^***^	(0.018)
Non-Active	0.061^***^	(0.013)	0.059^***^	(0.013)	0.078^***^	(0.013)
Manual worker	0.022	(0.017)	0.021	(0.017)	0.042^*^	(0.016)
Self-employed & small employers	−0.018	(0.023)	−0.018	(0.023)	0.011	(0.023)
Routine non-manual workers	0.017	(0.014)	0.016	(0.014)	0.029^*^	(0.014)
Denomination (Non-believer = ref)						
Catholic	−0.037^**^	(0.012)	−0.036^**^	(0.012)	−0.004	(0.012)
Other Christian	−0.016	(0.013)	−0.017	(0.013)	0.017	(0.013)
Other denomination	−0.000	(0.025)	−0.003	(0.025)	0.005	(0.025)
Urbanization (Rural = ref)						
Small or middle sized town	−0.002	(0.010)	−0.003	(0.010)	−0.006	(0.010)
Large town	−0.009	(0.011)	−0.011	(0.011)	−0.022^*^	(0.011)
Constant	2.209^***^	(0.035)	2.203^***^	(0.035)	2.433^***^	(0.036)
sd(CountryYear)	0.041^***^	(0.008)	0.042^***^	(0.008)	0.033^***^	(0.007)
sd(Residual)	0.604^***^	(0.004)	0.604^***^	(0.004)	0.590^***^	(0.004)
Observations	38,009		38,009		38,009	
*Notes*: *p < 0.05, **p < 0.01, ***p < 0.001 (two-tailed). Sources data: EB 77.4 & 83.4 (individual-level), MIPEX (national-level policies), Eurostat (GINI and percentage foreign born), World Bank (GDP); n-individuals: 38,009, n-country-years: 52. All macro variables are lagged (one year) and centered on the grand mean.						

Table 6 (Continued). Support for monitoring recruitment procedures (model 4 to 5).				
	Model 4		Model 5	
	b	se	b	se
*Individual-level variables*				
Gender (Male = ref)	0.098^***^	(0.008)	0.083^***^	(0.008)
Ethnic minority	0.030	(0.020)	0.040^*^	(0.020)
Religious minority	−0.019	(0.021)	−0.023	(0.021)
Sexual minority	−0.053	(0.037)	−0.064	(0.037)
Disability minority	0.029	(0.025)	0.018	(0.025)
Other minority	−0.050	(0.028)	−0.056^*^	(0.028)
Experienced discrimination	0.050^***^	(0.011)	0.017	(0.011)
Outgroup prejudice	−0.049^***^	(0.002)	−0.053^***^	(0.002)
Intergroup contact	0.027^***^	(0.003)	0.021^***^	(0.003)
Left–right placement	−0.022^***^	(0.002)	−0.018^***^	(0.002)
Missing left–right placement	0.005	(0.011)	0.011	(0.011)
*National-level conditions*				
Income inequality (GINI)	0.021^**^	(0.007)	0.024^***^	(0.007)
Antidiscrimination policies	0.005^**^	(0.001)	0.004^*^	(0.001)
Labor market integration policies	0.000	(0.001)	−0.000	(0.001)
Belief prevalence discrimination			0.116^***^	(0.006)
Belief unequal opportunities hiring			0.018^***^	(0.002)
*Individual-level control variables*				
Age	−0.002^***^	(0.000)	−0.001^***^	(0.000)
Education	0.002	(0.001)	0.002	(0.001)
Social position (service class = ref)				
Unemployed	0.072^***^	(0.018)	0.070^***^	(0.018)
Non-Active	0.079^***^	(0.013)	0.079^***^	(0.013)
Manual worker	0.042^*^	(0.016)	0.047^**^	(0.016)
Self-employed & small employers	0.011	(0.023)	0.016	(0.023)
Routine non-manual workers	0.029^*^	(0.014)	0.032^*^	(0.014)
Denomination (Non-believer = ref)				
Catholic	−0.005	(0.012)	0.006	(0.012)
Other Christian	0.016	(0.013)	0.026^*^	(0.013)
Other denomination	0.004	(0.025)	0.017	(0.025)
Urbanization (Rural = ref)				
Small or middle sized town	−0.006	(0.010)	−0.007	(0.010)
Large town	−0.022^*^	(0.011)	−0.028^**^	(0.011)
*National-level control variables*				
GDP *per capita*	−0.002	(0.002)	−0.001	(0.002)
Foreign-born population (%)	0.009	(0.005)	0.007	(0.005)
Constant	2.429^***^	(0.034)	2.106^***^	(0.036)
sd(CountryYear)	0.022^***^	(0.005)	0.021^***^	(0.004)
sd(Residual)	0.590^***^	(0.004)	0.580^***^	(0.004)
Observations	38,009		38,009	

*Notes*: ^*^p < 0.05, ^**^p < 0.01, ^***^p < 0.001 (two-tailed). Sources data: EB 77.4 & 83.4 (individual-level), MIPEX (national-level policies), Eurostat (GINI and percentage foreign born), World Bank (GDP); n-individuals: 38,009, n-country-years: 52. All macro variables are lagged (one year) and centered on the grand mean.

**Table 7:** Support for monitoring workforce composition (model 1 to 3).

	Model 1		Model 2		Model 3	
	b	se	b	se	b	se
*Individual-level variables*						
Gender (Male = ref)	0.129^***^	(0.009)	0.126^***^	(0.009)	0.110^***^	(0.009)
Ethnic minority	0.060^**^	(0.022)	0.047^*^	(0.022)	0.041	(0.022)
Religious minority	−0.007	(0.022)	−0.017	(0.022)	−0.010	(0.022)
Sexual minority	−0.001	(0.039)	−0.015	(0.039)	−0.036	(0.039)
Disability minority	0.072^**^	(0.027)	0.054^*^	(0.027)	0.053^*^	(0.027)
Other minority	−0.012	(0.030)	−0.027	(0.030)	−0.031	(0.030)
Experienced discrimination			0.066^***^	(0.011)	0.067^***^	(0.011)
Outgroup prejudice					−0.041^***^	(0.002)
Intergroup contact					0.014^***^	(0.003)
Left–right placement					−0.021^***^	(0.002)
Missing left–right placement					0.034^**^	(0.012)
*Individual-level control variables*						
Age	−0.003^***^	(0.000)	−0.003^***^	(0.000)	−0.002^***^	(0.000)
Education	−0.004^**^	(0.001)	−0.004^**^	(0.001)	−0.007^***^	(0.001)
Social position (service class = ref)						
Unemployed	0.101^***^	(0.019)	0.092^***^	(0.019)	0.099^***^	(0.019)
Non-Active	0.093^***^	(0.014)	0.091^***^	(0.014)	0.101^***^	(0.014)
Manual worker	0.066^***^	(0.017)	0.066^***^	(0.017)	0.077^***^	(0.017)
Self-employed & small employers	−0.005	(0.025)	−0.006	(0.025)	0.015	(0.024)
Routine non-manual workers	0.030^*^	(0.015)	0.030^*^	(0.015)	0.037^*^	(0.015)
Denomination (Non-believer = ref)						
Catholic	0.012	(0.013)	0.013	(0.013)	0.040^**^	(0.013)
Other Christian	0.021	(0.014)	0.020	(0.014)	0.048^***^	(0.014)
Other denomination	0.066^*^	(0.026)	0.062^*^	(0.026)	0.068^**^	(0.026)
Urbanization (Rural = ref)						
Small or middle sized town	0.004	(0.010)	0.003	(0.010)	−0.000	(0.010)
Large town	0.001	(0.011)	−0.002	(0.011)	−0.010	(0.011)
Constant	2.026^***^	(0.039)	2.018^***^	(0.040)	2.244^***^	(0.042)
(CountryYear)	0.056^***^	(0.011)	0.056^***^	(0.011)	0.050^***^	(0.010)
sd(Residual)	0.664^***^	(0.005)	0.664^***^	(0.005)	0.654^***^	(0.005)
Observations	38,009		38,009		38,009	
*Notes*: *p < 0.05, **p < 0.01, ***p < 0.001 (two-tailed). Sources data: EB 77.4 & 83.4 (individual-level), MIPEX (national-level policies), Eurostat (GINI and percentage foreign born), World Bank (GDP). n-individuals: 38,009, n-country-years: 52. All macro variables are lagged (one year) and centered on the grand mean.						

Table 7 (Continued). Support for monitoring workforce composition (model 4 to 5).				
	Model 4		Model 5	
	b	se	b	se
*Individual-level variables*				
Gender (Male = ref)	0.110^***^	(0.009)	0.096^***^	(0.009)
Ethnic minority	0.041	(0.022)	0.049^*^	(0.021)
Religious minority	−0.010	(0.022)	−0.016	(0.022)
Sexual minority	−0.036	(0.039)	−0.048	(0.039)
Disability minority	0.053^*^	(0.027)	0.041	(0.027)
Other minority	−0.031	(0.030)	−0.040	(0.030)
Experienced discrimination	0.067^***^	(0.011)	0.031^**^	(0.011)
Outgroup prejudice	−0.041^***^	(0.002)	−0.044^***^	(0.002)
Intergroup contact	0.014^***^	(0.003)	0.008^**^	(0.003)
Left–right placement	−0.021^***^	(0.002)	−0.018^***^	(0.002)
Missing left–right placement	0.033^**^	(0.012)	0.038^**^	(0.012)
*National-level conditions*				
Income inequality (GINI)	0.030^***^	(0.009)	0.032^***^	(0.009)
Antidiscrimination policies	0.004^*^	(0.002)	0.003	(0.002)
Labor market integration policies	−0.001	(0.002)	−0.001	(0.001)
Belief prevalence discrimination			0.131^***^	(0.007)
Belief unequal opportunities hiring			0.009^***^	(0.002)
*Individual-level control variables*				
Age	−0.002^***^	(0.000)	−0.002^***^	(0.000)
Education	−0.006^***^	(0.001)	−0.007^***^	(0.001)
Social position (service class = ref)				
Unemployed	0.099^***^	(0.019)	0.099^***^	(0.019)
Non-Active	0.101^***^	(0.014)	0.103^***^	(0.014)
Manual worker	0.077^***^	(0.017)	0.082^***^	(0.017)
Self-employed & small employers	0.015	(0.024)	0.021	(0.024)
Routine non-manual workers	0.038^*^	(0.015)	0.041^**^	(0.015)
Denomination (Non-believer = ref)				
Catholic	0.038^**^	(0.013)	0.048^***^	(0.013)
Other Christian	0.048^***^	(0.014)	0.057^***^	(0.014)
Other denomination	0.067^*^	(0.026)	0.079^**^	(0.026)
Urbanization (Rural = ref)				
Small or middle sized town	−0.000	(0.010)	−0.001	(0.010)
Large town	−0.010	(0.011)	−0.015	(0.011)
*National-level control variables*				
GDP *per capita*	0.000	(0.003)	0.001	(0.003)
Foreign-born population (%)	0.004	(0.007)	0.002	(0.006)
Constant	2.243^***^	(0.039)	1.918^***^	(0.041)
sd(CountryYear)	0.037^***^	(0.008)	0.035^***^	(0.007)
sd(Residual)	0.654^***^	(0.005)	0.645^***^	(0.005)
Observations	38,009		38,009	

*Notes*: ^*^p < 0.05, ^**^p < 0.01, ^***^p < 0.001 (two-tailed). Sources data: EB 77.4 & 83.4 (individual-level), MIPEX (national-level policies), Eurostat (GINI and percentage foreign born), World Bank (GDP). n-individuals: 38,009, n-country-years: 52. All macro variables are lagged (one year) and centered on the grand mean.

#### 4.2.1 Which citizens are more likely to support workplace diversity policies?

In Model 1 in Tables 5–7, we test the notion that people are more inclined to support workplace diversity policies if that is in their own interest because they belong to a (potential) target group, based on either their gender or self-identification as a member of a minority group. The results reveal, first of all, that women show significantly more support for all three types of workplace diversity policies than men. This supports *hypothesis 2a*. Likewise, we see that support for all types of diversity policies is significantly higher among people who regard themselves as a member of an ethnic minority group. Our results provide little evidence to support the idea that belonging to other minorities (religious, sexual, or other minorities) plays a similar role. The one exception is belonging to a minority group based on disability, which is related to significantly higher levels of support for monitoring the workforce composition, but is not significantly related to support for diversity training or monitoring recruitment procedures. To summarize, these results provide support for *hypothesis 2b* when it comes to the role of ethnic minority group membership and – to a lesser extent – minority group membership based on disability, but not for membership of other minority groups.

Another way to assess the role of self-interest is by examining how personal experiences with discrimination are related to support for workplace diversity policies, which we do in Model 2. The results show that support for all three types of workplace diversity policies is clearly and significantly higher among people who have personally experienced discrimination compared to those who have not. This supports *hypothesis 3*. As expected, after controlling for experienced discrimination in Model 2, the coefficient for belonging to an ethnic minority group clearly decreases in size compared to the previous model. This indicates that the higher level of support among ethnic minority members is partly due to the greater likelihood that they have personally experienced discrimination. Indeed, our data show that 43% of those who consider themselves a member of an ethnic minority group have personally experienced discrimination, compared to 17% amongst the majority population. Furthermore, the coefficient for gender remains fairly consistent when we control for experienced discrimination. Our data also show that there is a relatively small difference between men (16%) and women (19%) in the extent to which they have personally experienced discrimination. These results indicate that mechanisms other than being the victim of discrimination likely play a more important role in explaining the observed gender gap in support for diversity policies.

In Model 3, we examine the role of outgroup prejudice, intergroup contact, and political left–right self-placement in shaping support for workplace diversity policies. The results for these three determinants are consistent across all three type of workplace diversity policies. Prejudice is clearly and significantly related to lower levels of support for workplace diversity policies. This is in line with *hypothesis 4.* The coefficients for prejudice remain equally significant in subsequent models, showing that the role of prejudice is evident also when taking other predictors into account. Likewise, intergroup contact is found to be associated with support for all three types of diversity policies. Having more intergroup contacts is significantly related to more support for workplace diversity policies, which provides clear support for *hypothesis 5.* Furthermore, political orientation is associated with support for each of the three types of diversity policies. The coefficients are in the expected direction – a stronger right-wing political orientation is related to less support for diversity policies – thus providing support for *hypothesis 6.* Interestingly, after adding prejudice, contact and political self-placement to the model, the coefficients for gender and belonging to an ethnic minority group clearly decreased in size. The latter even dropped slightly below the threshold for statistical significance in this model. This indicates that the relationships between policy support and gender or ethnic minority group membership partly run through prejudice, contact and political self-placement. Note that the size of the coefficients for experienced discrimination in Models 2 and 3 are very similar. This underscores that personal experiences with discrimination are an important factor for explaining public support for workplace diversity policies, even when taking into account political ideology, prejudice and intergroup contact.

Finally, some results regarding the individual-level control variables deserve mention. Interestingly, respondents’ education level is differently related to public support for the three types of workplace diversity policies (see Models 1). A higher educational level is related to *more* support for diversity training and for monitoring recruitment procedures, but to *less* support for monitoring workforce composition. In all three cases, the coefficient for education is statistically significant. Results indicate that the positive relationship between education and support for diversity training and for monitoring recruitment procedures is partly explained by the lower level of prejudice and higher level of intergroup contact among the higher educated. In Model 3, controlling for prejudice and intergroup contact, the positive relationship between education and public support is strongly reduced (see Table 5 for support for diversity training) or even no longer significant (see Table 6 for support for monitoring recruitment procedures). By contrast, the negative coefficient for education regarding support for monitoring workforce composition increased after controlling for prejudice and intergroup contact (see Table 7, Model 3). We return to these effects of education in our discussion. Regarding respondents’ social position, we consistently found more support for all three types of workplace diversity policies among those who are currently not active at the labor market.^11^ In addition, those who are unemployed showed higher support for two out of three types of policies (i.e., monitoring recruitment procedures and monitoring workforce composition). We hardly found any significant results regarding the other social position categories. Furthermore, we consistently found that the older respondents are, the lower their support for workplace diversity policies. Regarding religious denomination, we found no strong and consistent differences between religious groups. Finally, there were no significant differences in public support between those living in rural, small or large towns.

#### 4.2.2 The role of macro-level conditions and sociotropic beliefs

In Model 4, country-level conditions were added to the analyses. Specifically, we added income inequality (GINI) and the two (MIPEX) policy indices to the model, whilst controlling for GDP *per capita* and the relative size of the foreign-born population. Results show that the level of income inequality in a country is significantly related to the extent to which inhabitants of that country support workplace diversity policies: the higher the income inequality in the country one lives in, the more one tends to support diversity initiatives. This is true for all three types of diversity policies, which provides clear support for *hypothesis 8.*

Regarding the role of national-level labor market mobility and anti-discrimination laws and policies, the results are more mixed. Labor market mobility laws and policies that foster migrant integration were only significantly related to support for diversity training, with support generally being higher in countries with more inclusive policies and laws. We found no significant associations with support for the other two workplace diversity policies. Antidiscrimination legislation and policies, by contrast, turned out to be significantly related to all three types of diversity policies. These conclusions hold when controlling for countries’ GDP and foreign-born population. Hence, we find partial support for *hypothesis 9*. With regard to the control variables at the contextual level (GDP and the share of foreign-born population), we note that we found mostly no significant relationships with public support for workplace diversity policies. Only the share of foreign-born population was significantly related to support for one type of policies: the higher the relative size of the foreign-born population, the stronger the public support for diversity training policies.

Finally, in Model 5, we assess the role of sociotropic beliefs regarding the prevalence of discrimination and unequal opportunities in the country one lives in. We find that both beliefs are consistently and significantly related to support for all three types of diversity policies. That is, in line with *hypothesis 7*, those who believe discrimination to be more widespread and hiring opportunities to be more unequal in their country tend to show higher levels of support for workplace diversity policies. Interestingly, after controlling for sociotropic beliefs, the sizes of the coefficients for intergroup contact clearly decrease, for all types of diversity policies. This indicates that the relationship between intergroup contact and policy support partly runs through individuals’ beliefs about the prevalence of discrimination and unequal opportunities in their country: having more intergroup contacts is related to stronger beliefs that discrimination and unequal opportunities exist, which in turn is related to higher levels of support. Similarly, after adding sociotropic beliefs, we see a slight decrease in the size of the coefficients for national antidiscrimination policies in Tables 5–7. This indicates that the higher public support for diversity policies in countries with more comprehensive antidiscrimination laws is partly explained by the fact that people in these countries are more often convinced that discrimination and unequal opportunities are prevalent in their society. That is, more extensive antidiscrimination policies seem to raise awareness about discrimination and inequality in society, which in turn boosts support for workplace initiatives that address these issues.

## 5 Conclusion and discussion

This study sought to shed light on patterns and determinants of public attitudes toward workplace diversity policies in Europe. We addressed three overarching questions, namely (1) to what extent is there public support for or opposition to three different types of workplace diversity policies in Europe, (2) which European citizens are more or less likely to support workplace diversity policies and why, and (3) how are national-level income inequality and labor market mobility and antidiscrimination laws and policies related to public support for workplace diversity policies?

### 5.1 Conclusion

Our findings underscore some key results from prior research on attitudes toward affirmative action and workplace diversity policies in the US, showing that public support for workplace diversity policies in Europe is – at least partly – shaped by the same factors. Specifically, in line with evidence from prior work on attitudes toward affirmative action policies (Bobo and Kluegel, 1993; Steeh and Krysan, 1996; Bobo, 1998; Harrison et al., 2006) and workplace diversity policies (Scarborough et al., 2019) in the US, our results revealed clear differences in public support for workplace diversity policies according to citizens’ gender and ethnic background. Women and people belonging to ethnic minority groups showed clearly higher average levels of support compared to men and ethnic majority members. Also consistent with findings from previous studies in the US on support for affirmative action policies (Dawson, 2001; Boeckmann and Feather, 2007; Kane and Whipkey, 2009; Vescio et al., 2016) and workplace diversity policies (Scarborough et al., 2019), we found that beliefs about the prevalence of discrimination and unequal opportunities in hiring in society are important factors shaping citizen’s attitudes toward workplace diversity policies. Finally, like prior studies in the US (Sniderman and Carmines, 1997; Scarborough et al., 2019), our results revealed a clear association between political orientation and public support, with those who are more left-leaning being significantly more supportive of workplace diversity policies than those who are more right-leaning.

Importantly, this study also goes beyond supporting previous research, yielding several crucial new insights regarding which individual-level and contextual-level conditions are associated with public support for workplace diversity policies in Europe. First, most studies in this field base their predictions on the role of gender, minority group membership and membership of other demographic groups that may benefit from diversity initiatives on theoretical considerations regarding self-interest or group-interest (c.f., Harrison et al., 2006; Avery, 2011; Scarborough et al., 2019; Bourabain and Verhaeghe, 2023). Our findings, however, paint a more nuanced picture. In the case of the role of ethnic minority group membership, our results indicate that the higher support among ethnic minority group members is indeed partly related to the fact that they have more often personally experienced discrimination than majority group members. Conversely, experiences with discrimination appear to play a much more modest role in accounting for the fact that women show higher levels of support for workplace diversity policies then men. This is in line with Robinson’s underdog thesis, which holds that, due to their underprivileged position in society, women tend to be more supportive of justice for other outgroups (Robinson, 1983). Importantly, our results also provide more information about other factors underlying gender difference in support. They show that, in part, women’s stronger support is related to the fact that, compared to men, they tend to be less prejudiced, have more intergroup contacts and *t* to be politically more left-leaning. Moreover, women’s higher levels of support partly stem from the fact that, compared to men, they are more likely to believe discrimination and unequal opportunities in hiring to be prevalent in their country. Still, when controlling for these other factors, clear and significant gender differences in support for workplace diversity measures persists. Future research may, therefore, delve deeper into the question which factors explain gender differences in support for workforce diversity policies.

Second, as a further test of the role of self-interest, we examined whether support for workplace diversity initiatives is higher among members of other minority groups. We did find some evidence of support being higher among those who consider themselves a member of a minority group based on a disability, which lends support to the notion that those who stand to gain from diversity policies are more supportive of these policies. However, we found no support for the notion that belonging to other minority groups (a religious, sexual or other minority group) significantly affects support for workplace diversity policies. This raises the question whether and why the self-interest logic does not seem to apply for these groups. One possible explanation is that workplace diversity policies, particularly regarding recruitment and selection processes, are often targeted toward visible minorities – like women, ethnic minorities, and people with a (visible) disability – and less so toward, for example, religious or sexual minorities. Consequently, especially people belonging to visible minorities might perceive workplace diversity policies as being in their benefit. That implies that for other types of workplace diversity policies – that were beyond the scope of this paper – one *may* find stronger support among religious and sexual minorities, if these are specifically targeted toward these groups (e.g., by facilitating prayer rooms or dietary rules, or peer support groups within the organization). Another possible explanation for this pattern of results is that some minority groups in our data are too small to allow for a meaningful test of our hypothesis about the role of minority group membership. Future research may shed more light on this, for example by examining support for a wider range of diversity initiatives, or by collecting data among (larger) samples of different minority groups.

Third, our study showed that mechanisms other than self-interest need to be taken into account to fully understand the factors driving attitudes toward workplace diversity measures. In this respect, our results yielded several key insights that are particularly relevant to our understanding of – and ability to leverage – support for diversity initiatives among members of advantaged groups. For example, by examining a broader range of *individual-level* factors than prior studies did, we were able to show that there are strong associations between the extent to which citizens are prejudiced and have intergroup contacts on the one hand and their level of support for workplace diversity initiatives on the other hand.

Fourth, and related to the previous point, another key contribution of this study was that it also derived and tested predictions about the extent to which levels of public support vary in accordance to *national-level* factors. Our results showed that countries’ level of income inequality is related to support for all three types of workplace diversity policies – with support being higher in countries with higher levels of income inequality. The same applies to people’s *perceptions* of the prevalence of discrimination and unequal opportunities in their country. Furthermore, we showed that national-level labor market mobility and antidiscrimination laws and policies play a role. Whilst labor market policies were only related to support for diversity training, stronger antidiscrimination laws and policies were associated with higher support for all three types of workplace diversity policies. Taken together, these results show that differences in public support for workplace diversity policies are partly driven by actual income inequality at the societal level, by citizens’ assessment of unequal opportunities and discrimination within their society, *and* by national-level laws and policies to fight unequal opportunities and discrimination. We therefore conclude that, to fully understand what explains public attitudes toward workplace diversity initiatives, future research should not only study the impact of individual-level factors, but also take into account contextual-level factors. Moreover, although studying the effects of *organizational*-level factors is crucial when considering support or resistance to workplace policies among employees or organizational leaders (i.e., within organization*s*), to obtain an accurate understanding of how contextual conditions shape attitudes toward workplace policies – both among the general public *and* within organizations – it is crucial to also take into account the *national* context.

Finally, we examined public support for three different types of workplace diversity policies and found less support for more preferential and prescriptive policies. This forms an important contribution to the literature because, so far, very few studies have systematically investigated the extent to which support varies across different types of policies (c.f., Scarborough et al., 2019; Bourabain and Verhaeghe, 2023). We found that public support was highest for diversity training, followed closely by monitoring recruitment procedures, whilst public support for monitoring workforce composition was clearly lower. Moreover, we revealed that in several instances, the factors shaping support are related to a different extent or even in a different way to support for different types of policies. This was the case, for example, for national-level labor market policies. We also found differential effects of citizens’ education across policies. In line with previous research that showed that higher educated tend to be more tolerant (e.g., Vogt, 1997) and have more positive attitudes toward outgroups (e.g., Coenders and Scheepers, 2003), we found that a higher educational level is associated with stronger support for two of the three workplace diversity measures, i.e., diversity training and monitoring recruitment procedures. By contrast, we found that a higher level of education is related to *less* support for monitoring workforce composition. A potential explanation for this finding is that the latter policy is the most preferential one and hence could be seen as violating the merit principle. The differential effect of education resonates with studies on affirmative action in the US which showed that education was related to more support for general principles of (racial) equality and opportunity enhancing programs but to *less* support for preferential treatment in hiring and promotion (for an overview see Schuman et al., 1997; Krysan, 2000). Some authors have therefore argued that higher educated individuals merely show a superficial commitment to equality, and are more sophisticated practitioners of a status quo ideology to defend their group interests (Jackman and Muha, 1984). Following this reasoning, Kane and Whipkey (2009) interpret the lower support among higher educated people for gender-related affirmative action in terms of a stronger endorsement among the higher educated of the individualistic ideology and meritocratic ideal that legitimates inequality. More research is needed to explore how the effects of individual demographic and ideological beliefs interact with support for different types of – and justifications for – workplace diversity policies.

### 5.2 Limitations and directions for future research

This study also has some limitations that merit attention. Based on these limitations *and* our conclusions, we identify several promising directions for future research. First, our repeated cross-sectional design does not allow us to draw *causal* conclusions about the individual and national-level correlates of public support for workplace diversity policies. In particular the relationship between national legislation and policies and public support is likely to be reciprocal. Still, our findings regarding the relationship between national legislation and policies (measured in the preceding year) and public support for workplace diversity policies are in line with the policy feedback theory (Mettler and Soss, 2004), which emphasizes the role of national-level legislation and policies for public agenda setting, enforcing social norms of equality and anti-discrimination, and increasing awareness of the need to improve inclusion and equal opportunities in organizations. Going forward, studies using either a panel design or experimental approach may shed more light on causality.

Additionally, in this study, we applied figures from the MIPEX project, which measure the extent to which national laws and policies aim to facilitate inclusion and participation of migrants or citizens with a migration background. Future studies could address the role of legislation and policies targeted toward other minority groups. Unfortunately, we are not aware of available large-scale cross-national data on such policies, but focusing on changing policies and public support within nations over time could also shed more light on the relationship between policies and public support.

Moreover, although the Eurobarometer data we used are, to our knowledge, the only existing cross-national survey data that contain measurements of public attitudes toward workplace diversity policies, these measurements do have some drawbacks. First, they capture support for a limited selection of workplace diversity policies. Yet, levels of public support do depend on the type of workplace diversity policy. Hence, future research may study support to a greater variety of workplace policies. Moreover, by directly comparing support for a wider range of workplace diversity initiatives, future studies could further our understanding of how much and why public support varies across different types of policies – for example testing more precise predictions on the extent to which workplace policies are prescriptive or preferential. Second, levels of support may also differ according to the target group of a policy. Some policies may be deemed acceptable when targeted at one group, but much less so when targeted at another group. Note, for example, that quota are an integral part of the debate in Europe regarding policies to increase the representation of woman on company boards (Rankin, 2022), whereas they are seldomly discussed as a way to increase the representation of other target groups (e.g., people with a migrant background) on boards or in organizations in general. Hence, further research may delve deeper into whether and why support varies depending on the type of policy *and* the target group. Third, the survey items we used to measure policy support contained a rather general description of each policy. This might (partly) explain why we found relatively high average levels of public support. Prior research on support for affirmative action has pointed out that “questions phrased very generally (…) coupled with a lack of knowledge may foster simple agreement” (Steeh and Krysan, 1996, p. 129). Imprecise questions may also increase social desirability bias – another factor that may partly explain the relatively high average levels of support we found – as the imprecise formulation avoids confronting respondents with potential costs or downsides of policies for some groups. Using more specific and detailed descriptions of workplace policies, rather than relying on people’s assumptions of what such policies entail, is thus likely to yield more accurate estimates of levels of public support (c.f., Scarborough et al., 2019) and shed more light on the conditions that boost or limit support for workplace diversity policies. Relatedly, future research may further explore the impact of the way employers communicate about or justify (i.e., frame) workplace diversity policies (Harrison et al., 2006; Scarborough et al., 2019; Jansen et al., 2021; Iyer, 2022).

Finally, future research could examine potential differences in support between the general public, employees, organizational leaders and other actors involved (e.g., job applicants), and how these depend on policy characteristics and the perceived need and justifications for such policies. A recent study on anonymous job application procedures in the Netherlands found striking differences in levels of support for anonymized procedures between managers, job applicants, and the general public, which were related to differences between these groups in the perceived need for and benefits and costs of such procedures (Blommaert and Coenders, 2023).

To conclude, we see this study as a starting point for more research on public attitudes toward various types of workplace diversity policies in Europe. Understanding the patterns and sources of public support and resistance for such policies is crucial for the successful diffusion and implementation of workplace diversity initiatives.

## Data availability statement

Publicly available datasets were analyzed in this study. This data can be found at: https://www.gesis.org/en/eurobarometer-data-service/survey-series/standard-special-eb/study-overview; https://ec.europa.eu/eurostat; https://2015.mipex.eu/download-pdf.

## Ethics statement

Ethical approval was not required for the study involving humans in accordance with the local legislation and institutional requirements. Written informed consent to participate in this study was not required from the participants or the participants’ legal guardians/next of kin in accordance with the national legislation and the institutional requirements.

## Author contributions

LB: Conceptualization, Data curation, Formal analysis, Investigation, Methodology, Writing – original draft, Writing – review & editing. MC: Conceptualization, Data curation, Methodology, Writing – original draft, Writing – review & editing.

## Footnotes

^1^For brevity, we will hereafter refer to such initiatives as “workplace diversity policies” with the understanding that we take this to include initiatives to increase diversity and inclusion, create more equal opportunities and reduce discrimination in the workplace. Some might summarize these as “diversity, equity, and inclusion (DEI) policies.” We acknowledge that the terminology used to refer to such policies differs across academic disciplines and national contexts and has evolved over time (see, for example, Iyer, 2022; Leslie and Flynn, 2022).

^2^Note, however, that studies that provide thorough, quantitative empirical evaluations to assess the effectiveness of workplace diversity interventions remain remarkably rare (c.f., Dobbin et al., 2015; Blommaert and Coenders, 2023; Chang et al., 2023).

^3^Note that Harrison et al. (2006) conflate both arguments, arguing that both the extent of preferential treatment and the extent to which policy initiatives limit decision makers’ discretion indicate violations of the merit principle. In other words, they assume that as policy prescriptiveness increases, so does the extent to which policies are preferential (merit-based norms are violated). Although in practice both elements (degree of preferential treatment and prescriptiveness) may often be intertwined, strictly speaking, policy initiatives may vary in both. For instance, in hiring practices, the discretion of managers can also be limited by non-preferential policy measures, such as in anonymous job application procedures or standardized application forms. Therefore, we discuss these features of policy initiatives separately.

^4^Policies that foster equal opportunities and inclusion can be regarded as a form of prosociality. Accordingly, public support for such policies may be motivated by human values such as self-transcendence (Schwartz, 1992) and driven by personal feelings of empathy and altruism. In this paper, we do not address such general underlying motivations for prosociality, but focus instead on specific intergroup attitudes, experiences with minority groups, and political-ideological beliefs that may particularly be relevant to understand public support for policies aimed to enhance opportunities and inclusion of disadvantaged groups.

^5^As indicated before, we refer to these as “workplace diversity policies,” but by this we mean policies to boost diversity, equality and inclusion and to reduce or prevent discrimination in the workplace. Although diversity takes center stage in the wording of these questionnaire items, they also refer to (groups at risk of) discrimination, and equal opportunities. Although diversity, equal opportunities, and discrimination are conceptually distinct, these questionnaire items lump them together. This mirrors the fact that – whilst policies to promote workplace diversity and inclusion, equal opportunities and anti-discrimination may have different rationales – in practice, organizations regularly implement a mix of policies and interventions, and it is often not explicitly communicated whether they do so to increase diversity, create equal opportunities, decrease discrimination, or a combination of those.

^6^We opted for a dichotomous variable as 82% did not feel discriminated against on any ground, 14% felt discriminated against based on one ground, and much smaller percentages felt discriminated against based on more than one ground (e.g., 3% on 2 grounds, 1% on 3 grounds).

^7^Although MIPEX is limited to migrant integration policies, we consider it a useful tool to compare national policies. Not only are ethnic, racial and national origin important dimensions of diversity and grounds of discrimination, but we also consider more inclusive policies with respect to migrant integration illustrative for the political salience of and extensiveness of inclusive and non-discriminatory policies in general.

^8^We used the MIPEX 2015 database rather than the 2020 database, because the former contains more comprehensive measures of each policy domain.

^9^The information on the macro-level independent variables refers to one year prior to the year the EB survey data were collected; e.g., the 2012 EB-survey data were matched to macro-level data from 2011.

^10^Using the ‘mixed’ command in Stata (version 17).

^11^We ran additional analyses in which we treated students (*n* = 2,834) as a separate category, rather than including them in the non-active category, to assess whether this changes our findings. Results show that that both non-actives *and* students show significantly higher support for all three types of diversity policies and other outcomes remain are unchanged.
